# Practical Departments

**Published:** 1903-02-21

**Authors:** 


### PRACTICAL DEPARTMENTS.

### A NOVEL FIRE-EXTINGUISHER: THE "EMERGENCY."

WEEKLY exhibitions of how to put out fire with the "Stevens Emergency Hand Fire Extinguisher" are being given in London, and one of the spots selected for the demonstration is an open space in Paul's Alley, leading out of Australian Avenue. The operator first piles up a slight structure of wood and shavings, saturates it with paraffin oil, and sets fire to it. The structure being about 10 feet high, and very inflammable, flames instantaneously rise to a considerable height, and the heat is intense. When the fire has well got hold of the pile, the top of the extinguisher is broken, and the fluid contained in it dashed on to the "root" of the flames. In about four seconds the fire is out and nothing but charred wood remains.

The extinguisher is a brass cylinder containing some "non explosive, non-corrosive and non-freezing" fluid, which is said to be harmless to flesh or fabric, and which, it is stated, may be swallowed with impunity. The cylinder is 21 inches long and 3 inches in diameter (household size, 2 inches) and weighs 6 pounds; it is to be hung on the wall, in the proportion of one dozen to every 5,000 square feet of flooring. It is said to be effective at 12 or 15 feet off, and not more than 14 "throws" should be necessary. The fluid generates a gas, the inventor states, which is absolutely free from anything noxious and from fumes liable to suffocate the person using it. It is an American invention, and is largely used in the United States and in the mill districts of Lancashire, where, it is stated, 7,000 tubes are in use, and 200 fires have been extinguished. The only London hospital mentioned as having yet adopted it is the German Hospital at Dalston, but the success and rapidity with which the fire in Paul's Alley was extinguished prove that the invention is worthy of a trial, and hospital authorities would do well to examine it for themselves. With permission of the Admiralty, demonstrations were recently given in Chatham and Portsmouth Dockyards, and on Ramsgate Sands—the last in a high gale—with great success.

The inventor, Mr. Stevens, says that after following carefully the details of the Colney Hatch fire, he believes it could easily have been extinguished at the outset by the use of two or three cylinders.

The factory is at 97 Whitworth Street, Manchester, and the London office is 680 Salisbury House, London Wall.

### THE UNIFORM SYSTEM OF ACCOUNTS.

A new edition of "The Uniform System of Accounts for Hospitals and Institutions," by Sir Henry Burdett, K.C.B., will shortly be issued by The Scientific Press, Limited. This system has now been adopted by all the principal hospitals and great institutions throughout the United Kingdom, and accepted by the Governments of the Colonies of Victoria and Queensland for introduction into every recognised institution within their jurisdiction.

